# Implementation of carbon pricing in an aging world calls for targeted protection schemes

**DOI:** 10.1093/pnasnexus/pgad209

**Published:** 2023-07-18

**Authors:** Peipei Tian, Kuishuang Feng, Heran Zheng, Klaus Hubacek, Jiashuo Li, Honglin Zhong, Xiangjie Chen, Laixiang Sun

**Affiliations:** Institute of Blue and Green Development, Shandong University, 180 Wenhua Xilu, Weihai, 264209, China; Department of Geographical Sciences, University of Maryland, 2181 LeFrak Hall, College Park, MD 20742, USA; The Bartlett School of Sustainable Construction, University College London, Gower Street, London, WC1E 6BT, UK; Integrated Research on Energy, Environment and Society, Energy and Sustainability Research Institute Groningen, University of Groningen, Nijenborgh 6, Groningen, 9747 AG, The Netherlands; Institute of Blue and Green Development, Shandong University, 180 Wenhua Xilu, Weihai, 264209, China; Institute of Blue and Green Development, Shandong University, 180 Wenhua Xilu, Weihai, 264209, China; Department of Geographical Sciences, University of Maryland, 2181 LeFrak Hall, College Park, MD 20742, USA; Department of Geographical Sciences, University of Maryland, 2181 LeFrak Hall, College Park, MD 20742, USA; School of Finance & Management, SOAS University of London, 10 Thornhaugh Street, Russell Square, London, WC1H 0XG, UK

**Keywords:** climate change mitigation, carbon pricing, carbon revenues recycling, aging

## Abstract

Understanding the impact of climate fiscal policies on vulnerable groups is a prerequisite for equitable climate mitigation. However, there has been a lack of attention to the impacts of such policies on the elderly, especially the low-income elderly, in existing climate policy literature. Here, we quantify and compare the distributional impacts of carbon pricing on different age–income groups in the United States, the United Kingdom, and Japan and then on different age groups in other 28 developed countries. We find that the elderly are more vulnerable to carbon pricing than younger groups in the same income group. In particular, the low-income elderly and elderly in less wealthy countries face greater challenges because carbon pricing lead to both higher rate of increase in living cost among low-income elderly and greater income inequality within the same age group. In addition, the low-income elderly would benefit less than the younger groups within the same income group in the commonly proposed carbon revenues recycling schemes. The high vulnerability of the low-income elderly to carbon pricing calls for targeted social protection along with climate mitigation polices toward an aging world.

**Significance Statement**

The global population is aging. Climate policy designs need to pay more attention to the elderly, especially the low-income elderly. This research finds that the low-income elderly and elderly in less wealthy countries face greater challenges to afford the increased cost of energy and other necessities of life resulting from the policy. Moreover, the low-income elderly would benefit less than the younger groups within the same income group in the commonly proposed carbon revenues recycling schemes. The results highlight the need for targeted social protection when implementing carbon pricing in an aging world.

## Introduction

Carbon pricing is a powerful financial instrument for carbon mitigation (1). An appropriate carbon price is expected to improve the competitiveness of renewable energy, promote low carbon production and consumption, and finance climate change mitigation efforts (2, 3). However, carbon pricing may also cause some negative effects, such as aggravating the inequality of distribution in some countries due to the greater economic losses of low-income groups in the short term (4–6). Such concerns about inequality may hinder the implementation of carbon pricing and discourage public engagement (7), even lead to social conflicts (8), such as the yellow vests movement in France, which protested against raising fuel tax. In this regard, understanding the impact of climate fiscal policies on vulnerable groups is the essential prerequisite for an equitable climate mitigation strategy (9).

The elderly, especially the low-income elderly, are generally considered as being among the most vulnerable groups due to their declining physical and mental faculties. The global population is aging (10). There are more than 1 billion people in the world aged above 60 years today, nearly twice that at the beginning of the 21st century (11). This number is projected to reach 2.1 billion by 2050 (10). The large and fast-growing vulnerable elderly population requires an urgent attention as part of global climate governance (12, 13). This population group is particularly vulnerable to climate change events, such as heatwaves and floods (14, 15). However, the impacts of climate mitigation policies such as carbon pricing on the elderly remain poorly understood.

Numerous studies have investigated the distributional effects of carbon pricing and showed the vulnerability of low-income groups (5, 6, 16–19), but few pay attention to the policy consequences of carbon pricing in an aging society. In particular, we know little about the situation of low-income elderly under carbon pricing. The low-income elderly may have to face the overlapping risks of lifestyle and income changes under carbon pricing. Unfortunately, there has been a lack of attention to this most vulnerable group in current carbon pricing literature and policies.

Compared with other age groups, the elderly have some particular lifestyles that increase their exposure to carbon pricing. For example, the elderly usually stay at home longer due to decreased mobility (20, 21) and consume more heating and cooling services (22, 23). For low-income elderly, they have very limited income sources, other than pensions and savings (24, 25). And more importantly, it is hard for them to improve their incomes due to social and physical barriers. Thereby, the elderly, especially the low-income elderly, are often exposed to poverty and much heavily rely on social protection programs (26). Specific lifestyles and financial situation make it harder for the elderly, especially the low-income elderly, to cope with extra costs (27). Therefore, a comprehensive understanding of the distributional effects of carbon pricing must include the distributional impact of carbon pricing on the elderly, especially the low-income elderly in an aging world.

Here, we quantify and compare the impacts of carbon pricing among different age–income groups in the United States, the United Kingdom, Japan, and then among age groups in other 28 developed countries (27 EU countries plus Australia), which have large shares of aging populations. Households are affected by two aspects when pricing carbon: direct expenditure increase in energy consumption and indirect expenditure increase in goods and services consumption because of the embodied carbon emissions across the production and transportation networks (28). Thereby, we use a global multiregional input–output model combined with detailed household expenditure survey (HES) data of different age and income groups to assess the direct and indirect impacts of carbon pricing (see Materials and methods). Given that expenditure is a comprehensive reflection of income, wealth, and lifestyle, the share of additional expenditure required for consumers to maintain their initial consumption level in the total is used to assess the impact of carbon pricing (4). The global carbon price is set at $40 per tonne of CO_2_, which is widely seen as an estimated lower bound consistent with the Paris goals (29). In this study, we first compare the impact of this carbon pricing regime on younger and elderly groups within the same income group in the United States, the United Kingdom, and Japan. These three countries are seen as the representatives or examples of the lifestyles in North America, Europe, and East Asia, and data for establishing the age–income paired groups are available there. Then, we expand the geographical scope of our research and investigated the impacts of carbon price on different age groups in all of these 31 northern countries. Last, we discuss the situation of the low-income elderly in the carbon revenues recycling schemes. By revealing the distributional impacts of carbon pricing between younger and elderly, our study provides insights into the policy consequences of carbon pricing on the elderly, which would facilitate equitable and sustainable climate mitigation policies in an aging world.

## Results

### Higher vulnerability of the elderly to carbon pricing

**Fig. 1.**
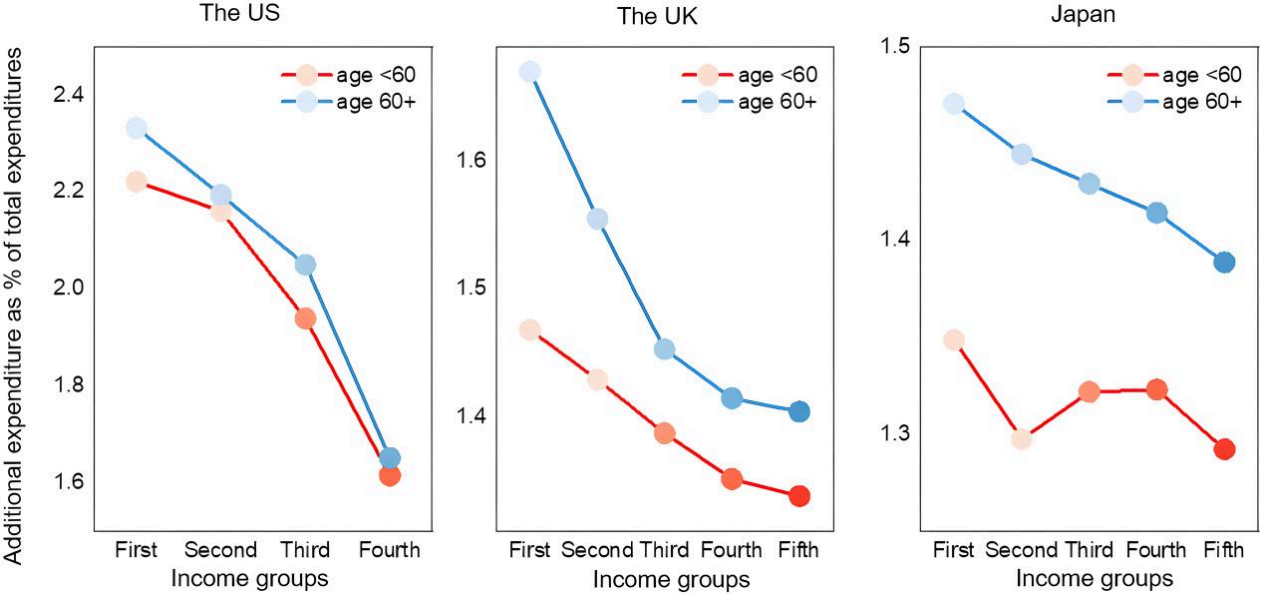
Impacts of a $40 per ton CO_2_ global carbon price on the combined income and age groups in the United States, the United Kingdom, and Japan, respectively. The order of income groups increases from the first (the lowest) to the fifth (the highest), corresponding to the shade from light to dark. Note that income groups in the United States are by specific amounts while in the United Kingdom and Japan are by quintiles.

As shown in Fig. 1, the impacts of carbon pricing on both younger and elderly high-income groups are relatively small, while the younger and elderly low-income groups have a higher ratio of the additional expenditure under carbon pricing. Carbon pricing has a regressive distribution impact in the United States, the United Kingdom, and Japan, which is in line with the previous research (6, 24, 25). But more importantly, we find that the ratio of additional expenditure of the elderly is consistently higher across income groups than that in the corresponding younger income group. For example, when the CO_2_ is pricing at $40 per ton, the shares of additional expenditure of the lowest-income elderly group are 2.35%, 1.67%, and 1.47% in the United States, the United Kingdom, and Japan, respectively, while these numbers are 2.22%, 1.46%, and 1.34% for lowest-income younger group in these three countries, respectively. The elderly group always needs to spend more than the younger group with the same income under carbon pricing, which implies that elderlies are more vulnerable to carbon pricing than younger groups for all income groups in the United States, the United Kingdom, and Japan. In addition, the regressivity of carbon pricing is stronger in the elderly groups compared with the younger groups in the United States, the United Kingdom, and Japan (Fig. 1), i.e. the gap in the impacts of carbon pricing between low-income and high-income groups on the elderly is bigger than the gap corresponding to the younger groups. The lowest-income elderly group is the most vulnerable group with the highest ratio of the additional expenditure among all groups.

**Fig. 2.**
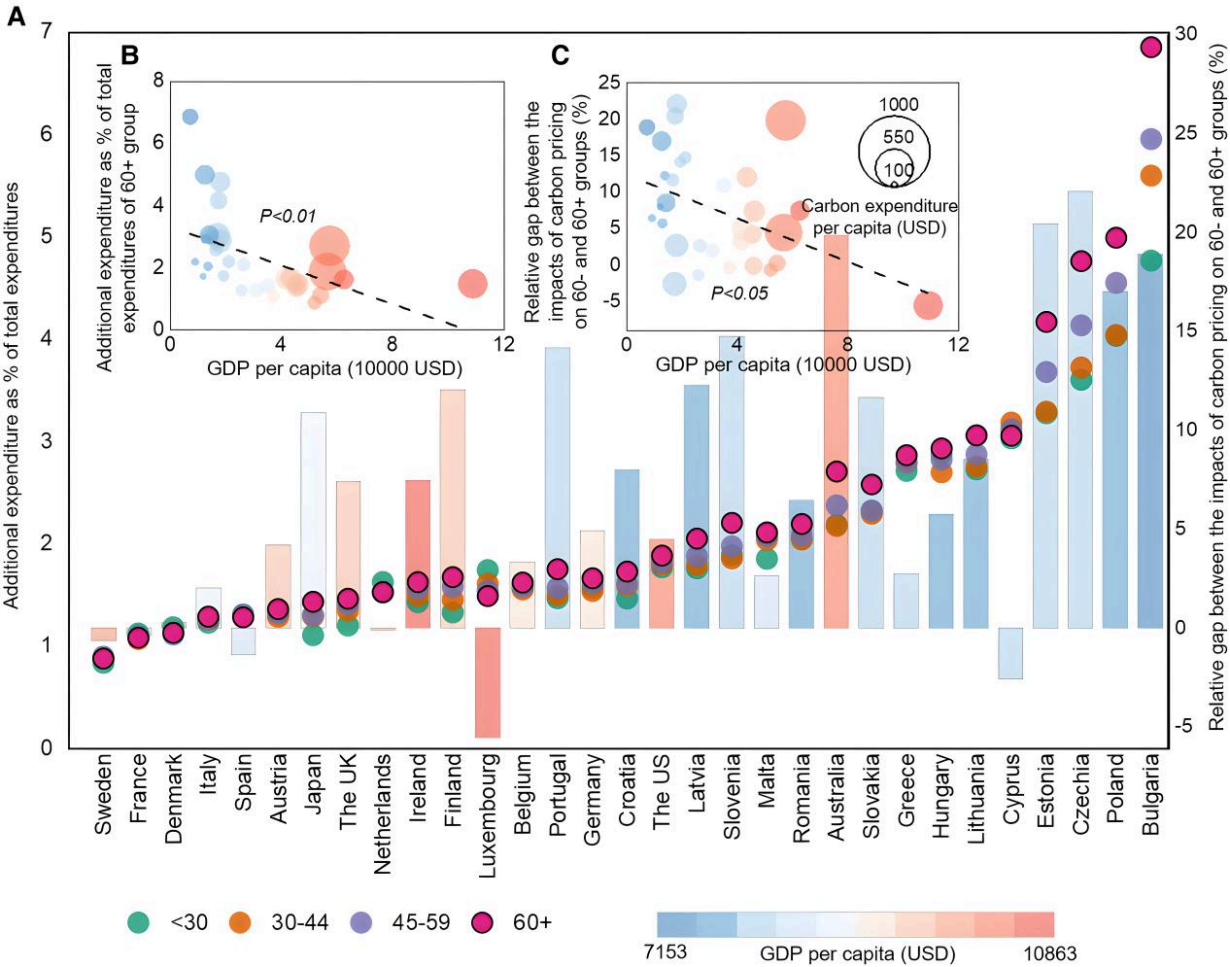
Impacts of $40 per ton CO_2_ global carbon price on different age groups in 31 countries. A) Impacts of carbon pricing on four age groups. Dots refer to the impacts of carbon pricing on four age groups (Left coordinate scale). Bars refer to the relative gap between the impacts of carbon pricing on 60− and 60+ groups (Right coordinate scale). Countries/regions are ranked by the national average impacts of carbon pricing. B) The correlation between GDP per capita and the absolute impacts of carbon pricing on the age 60+ group. C) The correlation between GDP per capita and the relative gap between the impact of carbon pricing on 60+ and 60− groups. The sizes of the dots in B and C refer to the per capita expenditure increase caused by carbon pricing. The bars in A and dots in B and C corresponds to the national GDP per capita, from the wealthiest countries (red) to the poorest countries (blue).

An extension of our research scope to different age groups in all 31 northern countries shows that the elderly group has the largest share of additional expenditure under a $40 per ton CO_2_ global carbon price than younger groups (dots in Fig. 2A). This unequal effect induced by carbon pricing between elderly and younger groups exists in almost all study countries, where the impact of carbon pricing on the expenditure of the elderly group is the highest among all age groups (dots in Fig. 2A). It is noteworthy that inequalities among age groups caused by carbon pricing in relatively less affluent countries such as Poland and Bulgaria are much larger than that in wealthy countries (e.g. the United States and Sweden). We further explore the relative gap between the impact of carbon pricing on elderly and younger groups (Fig. 2A and C). The relative gap equals the absolute difference between the burden rate of younger (60−) and elderly (60+) groups divided by the burden rate of younger (60−) group. For example, if the burden rates of younger (60−) and elderly (60+) groups are 1 and 1.5% under carbon pricing, respectively, the relative gap is (1.5–1%)/1% = 50%. We find that the relative gap between the impact of carbon pricing on elderly and younger groups is generally <10% in affluent countries, while those in less wealthy countries (mainly Eastern EU countries) are usually over 20%. A correlation analysis of this relative gap on per capita GDP shows a negative relationship at the 0.05 significant level (Fig. 2C). Therefore, it can be concluded that the elderly in less wealthy countries have a higher vulnerability to carbon pricing, both in relative and absolute terms (Fig. 2B and C).

### High share of additional expenditures for housing energy of the elderly under carbon pricing

**Fig. 3.**
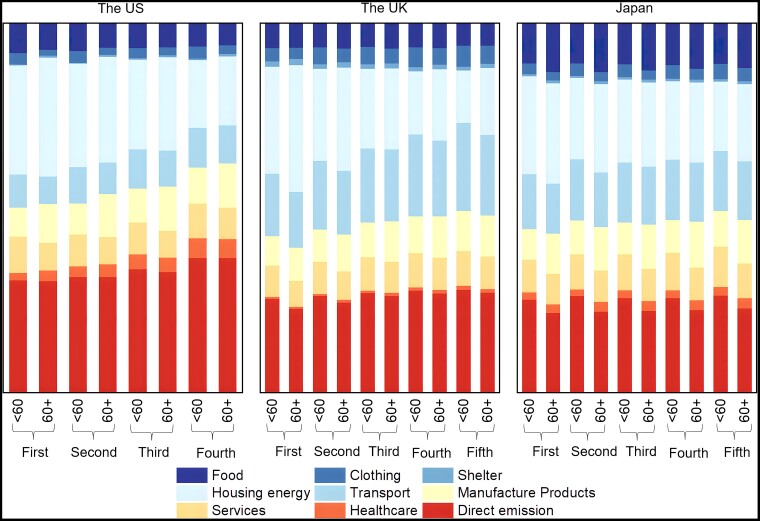
Sectoral structure of additional expenditures under $40 per ton CO_2_ global carbon price for income–age groups in the United States, the United Kingdom, and Japan, respectively. Income is ordered increasingly from the first (the lowest) to the fifth (the highest) levels. Note that income grouping in the United States is by specific amounts while in the United Kingdom and Japan is by quintiles.

The structure of additional expenditure caused by carbon pricing varies greatly with both income and age, with obvious difference across age groups in the same income group (Fig. 3). For example, the cost of carbon emissions from the housing energy and direct energy use is the main source of additional expenditure for all age and income combined groups for most countries/regions under the carbon pricing regime (Figs. 3 and S3). However, we find that the share of additional expenditure on housing energy in the total additional expenditure is higher for the elderly than the younger groups under global carbon price for all income groups. Compared with the younger, elderly are more likely to stay at home longer and need more stable cooling as well as heating due to their high sensitivity to ambient temperature change (20, 23). Meanwhile, smaller family sizes (30) and old houses lacking energy-saving renovation (31) also lead to a lower energy utilization efficiency in elderly households. A previous study in EU countries found that elderly households tend to have lower household electricity efficiency (32). Energy-dependent lifestyles and lower energy use efficiency of the elderly group usually result in a higher share of additional expenditure of housing energy in the total under the carbon pricing regime, which is more obvious in low-income elderly households. Thereby, the low-income elderly have a high share of additional expenditure of housing energy in the total additional expenditure, compared with high-income elderly (Fig. 3).

### The disadvantaged situation of the low-income elderly in the carbon revenues recycling schemes

**Fig. 4.**
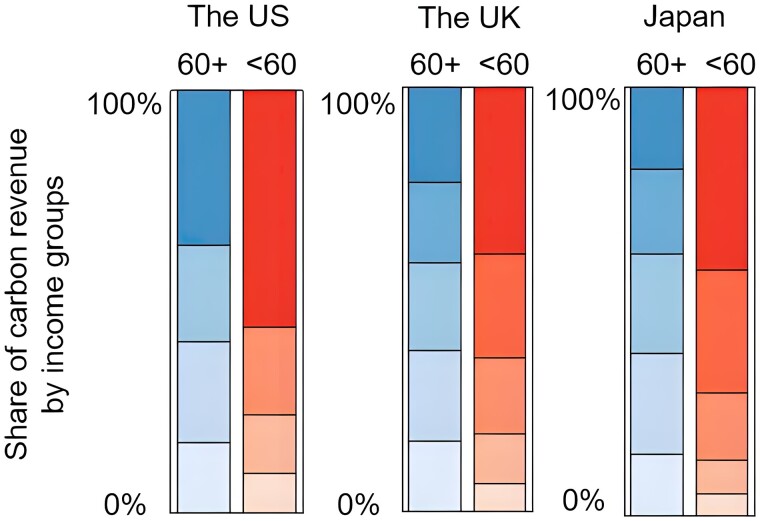
Shares of carbon revenue contribution for different income groups between the elderly (60+) and younger (60−) groups in the United States, the United Kingdom, and Japan, respectively. The shade from light to dark corresponds to the income groups from the first (the lowest) to the fifth (the highest).

We find that the lower-income groups in the elderly group contribute more to carbon revenue than the counterpart in the younger group (Fig. 4). For the elderly, the lowest two income groups in combination contribute 41, 40, and 37% to the total carbon revenue of all the elderly in the United States, the United Kingdom, and Japan, respectively, while the corresponding shares are 24%, 22%, and 13% in the younger group of the three countries. Meanwhile, the highest-income group among the elderly contributes less carbon revenues (37%, 22%, and 19%) compared with the high-income younger groups (56%, 42%, and 39%) in the United States, the United Kingdom, and Japan, respectively. Two reasons drive this: a higher population share of lower-income people and a higher additional expenditure caused by carbon pricing in the elderly group. In other words, the cost of carbon mitigation by carbon pricing is disproportionally born by lower-income households in the elderly group, thus departing from the goal of equitable and sustainable climate change mitigation.

Given the high vulnerability of the low-income elderly group to carbon pricing, the protection of the low-income elderly group is necessary. Previous studies usually pointed out that the carbon revenues recycling scheme is an effective option to reduce inequality and protect vulnerable groups (16, 27). We devise four scenarios to discuss the effect of carbon revenue rebates: (i) No rebate scenario, where there is no additional rebate for each group. (ii) Average rebate scenario, where carbon revenues are rebated to everyone equally. This equal per capita basis of rebate has received great attention and high expectation in the literature (33–37). (iii) Poverty rebate scenario, where people in the lower-income group can get 20% more refunds in reference to average rebate scenario. (iv) Elderly poverty rebate scenario, where the lower-income elderly can get 20% more refunds in reference to the poverty rebate scenario.

**Fig. 5.**
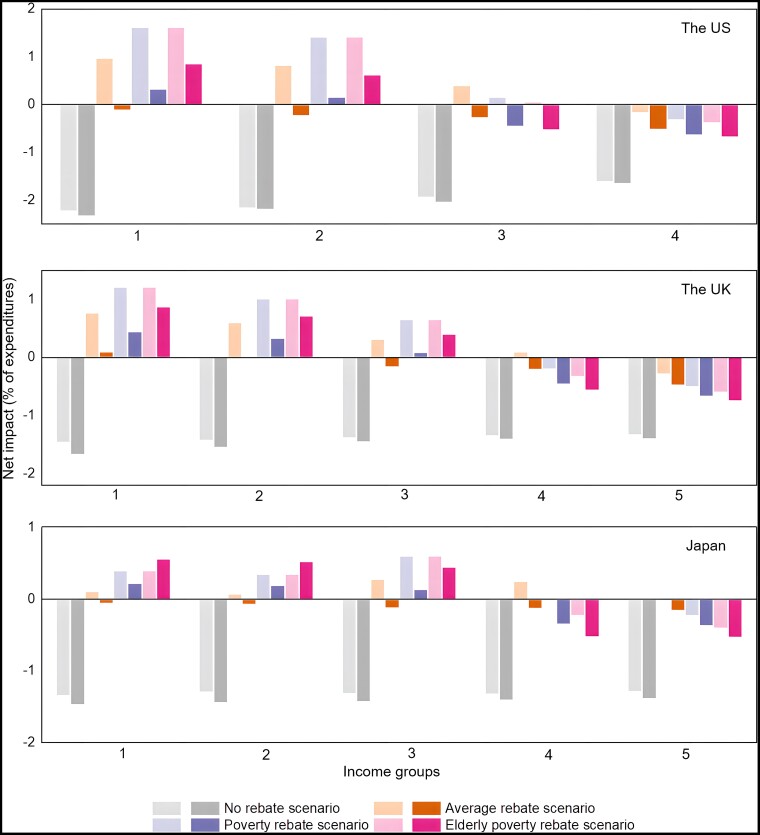
Net impacts of carbon revenues rebate scenarios on different age and income groups in the United States, the United Kingdom, and Japan, respectively. The light and dark shade refers to younger (60−) and elderly (60+) groups, respectively.

The younger groups except for the highest-income group would benefit from the average rebate scenario (light brown bars in Fig. 5), while no elderly group can get positive impacts from this scheme in the United States and Japan (dark brown bars). This indicates that all elderly groups, including the lower-income elderly group, are the net contributors in this average rebate scheme. In the United Kingdom, only one group of the elderly has net benefits. It can be found that the average rebate of carbon revenues ignores to a large extent the most vulnerable group—the low-income elderly. Although the lower-income elderly groups can get positive impacts under the poverty rebate scenario, their potential benefits (dark blue bars) are far less than those for lower-income younger groups (light blue bars). Finally, the elderly poverty rebate scenario has a more positive impact than both the average rebate and poverty rebate scenarios (red bars). All the lower-income groups are the beneficiaries under this recycling scheme, and the higher income groups are contributors.

## Discussion

Carbon pricing has been widely regarded as a necessary and effective policy tool in reducing carbon emissions. Its distributional impacts on vulnerable groups are an important issue for fair and efficient climate change mitigation. Our simulations show that the elderly are more vulnerable to carbon pricing than younger groups in each of the five income groups. The low-income elderly and elderly in less wealthy countries face greater challenges due to the disproportional burden of additional expenditure and increasing income inequality across age groups under the carbon pricing regime. We also find that the low-income elderly are at a disadvantage under the popularly proposed carbon revenues recycling schemes. Therefore, targeted schemes capable of preventing the low-income elderly group from disproportional burden of additional expenditure under the carbon pricing regime are necessary for protecting the vulnerable low-income elderly groups.

The rapidly aging population and deteriorating climate are double challenge to sustainable development in this century. The elderly group may suffer higher carbon mitigation costs in the transition to a low carbon world. Our findings reveal the vulnerability of the elderly in implementing carbon mitigation policies such as carbon pricing, which may lead to adverse consequences against the goal of equitable and sustainable climate change mitigation. For example, older people may have more difficulties to move away from the relatively more carbon insensitive consumption of necessities, which may weaken the effectiveness of carbon prices in an aging society. A previous study of main developed countries found that expenditures of the elderly group were more rigid and necessary, rather than a luxury attribute (12), which implies that the additional expenditure caused by carbon pricing may have very limited impacts on the change of lifestyle of the elderly (38, 39). Thereby, the elderly group may have more difficulties to turn to greener lifestyles driven by carbon pricing alone. With the rapid increase of the elderly population in the future, the vulnerability of the elderly to carbon pricing may weaken the effectiveness of the carbon pricing on carbon mitigation.

Moreover, a higher share of additional expenditure in the total as triggered by carbon pricing and a less flexible lifestyle may lead to additional adverse impacts at an older age, especially when the poverty rate of the elderly is relatively high (40). The poor elderly, with limited income sources, e.g. fixed government pensions and small plus shrinking savings (24, 25), may have more difficulty to cope with additional expenditure caused by carbon pricing, which may increase the risk of poverty for the elderly. This risk may be more severe in less wealthy countries, where carbon pricing has a higher impact on the expenditure of the elderly and the elderly meanwhile suffer higher inequality caused by carbon pricing. It is particularly the case of some eastern European countries where a sound pension system is absent and the low-income elderly do not have sufficient savings (41). For instance, nearly a quarter of Bulgarian pensioners received the minimum payment (only $82 per month) in 2016 (42). Carbon pricing would even worsen the situation of those low-income elderly groups, who contribute a larger share of their income to the carbon revenue in comparison with low-income younger group. In addition, the low-income elderly group usually has lower level of risk resistance and is more dependent on government assistance (43). They also face multiple physical, financial, and social barriers and thus may be at risk of climate mitigation policy marginalization. Hence, additional protections of government policy are necessary to prevent adverse impacts of carbon pricing on the low-income elderly.

Carbon revenues recycling schemes have been considered to be a great option to protect vulnerable groups. Previous studies of the schemes have included low-income groups with the aim to reduce the regressive impacts of the carbon pricing (44). However, there has been a lack of attention to the unfavorable situation of the low-income elderly group, which may undermine the mission of these schemes. The evaluation of climate change mitigation strategies has shown an increasing recognition that vulnerable groups must be identified and their vulnerability must be appropriately addressed by national government policy (45). This study further highlights the importance of combining carbon revenues recycling schemes with provision of old-age pensions and subsidies in promoting equitable and sustainable carbon pricing policies. It is also worth noting that in the increasingly digitalized social administration systems, it is becoming easier to identify and reach low-income elderly groups.

Many nations aspire a transition to the low carbon production and consumption. But it is insufficient to simply put the cost burden of this transition on the consumers. There may also be opportunities to address elderly protection and carbon mitigation in conjunction by supporting low-income elderly households with energy efficiency measures and thus reducing the impact of carbon pricing on them while mitigating carbon emissions (31, 46). In addition, renewable energy transition and heating electrification are also key to reducing the inequality impacts of carbon pricing and enhancing social support given that the elderly people are more dependent on energy.

To sum up, our analysis reveals that elderly people, especially low-income elderly and elderly in less wealthy countries, are at a potentially troubling disadvantage in the implementation of carbon pricing scheme. Our finds suggest the need for a clearer understanding between the welfare of low-income elderly and climate change mitigation in today's aging society.

## Materials and methods

### Distributional impact analysis using multiregional input–output analysis

The multiregional input–output (MRIO) approach has been widely used to estimate the distributional impacts of energy subsidies, carbon tax, and carbon pricing on different household consumption groups (5, 16–18, 47, 48). One great virtue of this method is that it can model both direct and indirect impacts of carbon pricing on household expenditures, i.e. including not just price rising for oil, gas, and other energy products, but also the price rising triggered by carbon emission costing to all household consumption items. Notably, the MRIO approach gives an upper-bound estimate of the short-term impact of carbon pricing on the price of consumption items. Compared with other carbon pricing effect simulation methods such as the computational general equilibrium model, MRIO cannot reflect the short-term substitutions of production factors in the economy. But it is more close to the intuition perceived by the public due to its transparency, making it a good policy evaluation tool for focusing on the social reaction to carbon pricing hikes (28, 49).

In this study, MRIO is applied to model the impacts of the carbon pricing shocks on four age groups (<30, 30–44, 45–59, and 60+) in the United States, the United Kingdom, Japan, and other 28 developed countries (27 EU countries and Australia). The classic Leontief demand model in the IO framework is adopted to allocate the carbon emission induced by households’ consumption (50), which can be expressed by


(1) x=Ax+y

alternatively,


(2) $$\Delta x=(I-A{)}^{-1}\Delta y$$

where *x* is the total output and *A* is the technical coefficient matrix of the economy. *y* is the final demand vector by sectors, including household consumption, capital formation, government expenditure, and exports. $(I-A{)}^{-1}$ is called the Leontief inverse matrix (or total requirement matrix), which shows the total production of each sector required to satisfy the final demand vector in the economy. Equation (2) means that a change in y (Δ*y*) leads to changes in total output (Δ*x*).

It should be noted that this study focuses on the direct and indirect changes in household consumption expenditures driven by carbon pricing. Therefore, Δ*y* reflects changes in household consumption only in this study.

Then, we estimate the expenditure increase per age group based on the MRIO model (16, 28, 51), as driven by the carbon pricing. The total expenditure increase of consumption is the sum of direct and indirect expenditure increase:


(3) $$C_{q}^{tot}=C_{q}^{dir}+C_{q}^{indir}$$

where $C_{q}^{tot}$, $C_{q}^{dir}$, and $C_{q}^{indir}$ are total, direct, and indirect expenditure increase of age group *q*, respectively.

To calculate the indirect expenditure increase $C_{q}^{indir}$, we add a row of the environmental multiplier *e* which is the cost increase per unit of sectoral output. Here, *e* is derived from the production cost increase in each economic sector due to the price of carbon emissions divided by the total sectoral output, mathematically:


(4) $$e=p\times f_{sctor}$$

where *p* is carbon price (cost per unit CO_2_ emission) and $f_{sctor}$ indicates the direct carbon emission intensity of sectors (CO_2_ emitted per unit of sector output).

Then, we obtain total indirect expenditure increase of consumption through linking the supply industries to the changes in the final consumption of age group *q* ($\Delta y_{q}$), which can be expressed by


(5) $$C_{q}^{indir}=e\times (I-A{)}^{-1}\Delta y_{q}$$

The direct expenditure increases $C_{q}^{dir}$ of carbon pricing shock on age group *q* can be calculated by multiplying the increase in household direct emissions of age group *q* ($F_{dir\_q}$) and the carbon price *p*, which be expressed as:


(6) $$C_{q}^{dir}=p\times F_{dir\_q}$$

Under the carbon price regime, household consumers have to spend more money to consume the same amount of goods and services. In this study, we choose the carbon payment burden rate to assess the impact of carbon pricing. Traditionally, two indicators are employed to measure the impact of carbon pricing in the literatures: (i) the absolute value of carbon payment and (ii) the carbon payment burden rate. The former signifies the absolute value of per capita cost for an individual's carbon emissions. However, as it fails to account for varying financial capabilities across different income groups, this absolute value is unable to reflect affordability. The rich and the poor, understandably, have divergent abilities to manage the same costs. Consequently, the latter measure, carbon payment burden rate, proves more pertinent in assessing the distributional implications of carbon pricing. This rate is defined as the ratio of the absolute carbon payment value to income and has been commonly used in both the literatures and government reports (4, 16, 51). Our study, conducted using the MRIO model, provides the estimates of the upper bounds of the short-term impact of price hikes on consumers before they have enough time to adjust their consumption behavior or take adaptive actions. Thereby, the absolute carbon payment value is the additional expenditure required for consumers to maintain their initial consumption level under carbon pricing. The literature points out that the expenditure is an inclusive reflection of income, wealth, and lifestyle; therefore, we use the total expenditure as the approximation of income (18, 52). Consequently, the share of additional expenditure $C_{q}^{tot}$ that consumers would need to uphold their initial consumption $y_{q}$ in the total is defined as the carbon payment burden rate under carbon pricing. This rate is used to measure the impact $I_{q}$ of carbon pricing on age group *q*:


(7) $$I_{q}=\frac{C_{q}^{tot}}{y_{q}}$$

### Data source and processing

We use the detailed household expenditure data and the global MRIO table of 2015 as well as carbon emission account from EXIOBASE to capture the heterogeneity of impacts of carbon pricing on different income and age groups. EXIOBASE is a global detailed environmentally extended MRIO database developed by harmonizing and detailing supply-use tables for a large number of countries, including 44 countries and 5 rest of the world regions (53). It provides a detailed sectoral classification with 200 products/163 sectors, with more than 1,000 environmental and social satellite accounts. In this study, EXIOBASE 3.7 which covers the year 2015 is used.

HES provide a comprehensive description of household and consumption characteristics, including household member parameters, detailed consumption items, and expenditures details. The HES data are collected from official statistics agencies (see Data availability). For HES data by age group in 31 countries, the HES data are presented by the age of the reference person (usually called household head, which is not a person nominally, but breadwinner of the household) of a household and the expenditures are presented by households. Grouping households according to the age of the household head is a common practice in the literatures given the household heads’ age has a significant impact on household expenditure patterns for their economic status. For HES data by combined income and age group in the United Kingdom, the United States, and Japan, the HES data are first divided into multiple income groups based on household income. Then, every income group is further divided into younger and elderly groups based on the age of household head (Supplemental Fig. S1). It is noteworthy that all HES data used in this study are nationally average published by official statistics agencies, rather than microlevel expenditure data.

All HES data adopt an expenditure nomenclature, the Classification of Individual Consumption by Purpose (COICOP), but the detailed classification varies in different countries, which is usually different from the classification of household demand in EXIOBASE table. Therefore, the RAS-based method is applied to bridge the difference in classifications between HES data (COICOP) and EXIOBASE (54). In the matching process, household consumption of EXIOBASE is set as the benchmark and HES data of all countries are converted into Euros with the exchange rate of 2015. We use the bridging matrix to link the expenditure items in the HES data to the household demand sectors in the EXIOBASE. In this reconciliation process, we calculate the final demand for each expenditure group in each country by multiplying the expenditure share of groups from the HES data with the household final demand from the EXIOBASE. It means that our analysis is consistently based on basic prices (producer price), and the information we retrieved from the HES data is the expenditure shares rather than the monetary values of expenditure. Finally, we get household final demand consistent with EXIOBASE classification of different income and age groups in study countries (Supplemental Fig. S1).

### Design options of carbon price

In this study, we model a carbon pricing regime of $40 per ton CO_2_ globally. This price level is widely seen as an estimated lower bound consistent with the Paris goals (29). Because the MRIO model is linear, our results can easily be scaled down or up to other carbon price levels (16, 28). In other words, the specific value of global carbon price will not affect the robustness of the results, i.e. the distributional impacts of carbon pricing between younger and elderly will not change under different global carbon prices. Except for the optimal global carbon price, many countries also have implemented national economy-wide carbon pricing schemes or schemes that target specific sectors in the early mitigation strategy (55), for example, a carbon price that focuses on the electricity sector. Therefore, we further discuss different design options of carbon pricing to ensure the robustness of our results (Supplementary Fig. S2).

### Carbon revenues recycling scenarios

Four carbon revenue recycling scenarios are developed in this study (16, 27, 56): (i) No rebate scenario, meaning no additional rebate for every group. (ii) Average rebate scenario, where carbon revenues are rebated to everyone equally. This equal per capita based rebate has been placed great attention and expectation in the literatures (33–37). (iii) Poverty rebate scenario, where people in the lower-income group can get 20% more refunds in reference to average rebate scenario. (iv) Elderly poverty rebate scenario, where the lower-income elderly can get 20% more refunds in reference to poverty rebate scenario.

## Limitations

Several limitations of this study are worth mentioning. First, as discussed in the method section, MRIO model is linear; our approach thus provides an upper-bound estimate of the short-term impact of carbon pricing on the price of consumption items, before the substitutions of production factors take place (16, 17, 28). Second, HES data by age groups are provided by the age of the reference person of a household (breadwinner) identified by the statistical agency of the studied countries, which may lead to uncertainties. For instance, younger people will be counted as a member of their parental household if they cohabit with their parents in most cases, unless young people are breadwinner (in very rare cases). Hence, the number of households with age <30 years may be underestimated in our study, as many youths may still cohabite with their parents. But we argue that this underestimation does not exert meaningful effects on the results of elderly groups for most of the studied countries, as the cases where children live with their parents aged 60+ could be very rare (12, 57). Moreover, the HES data provided by the National Bureau of Statistics are categorized into specific age groups (e.g. EU27 with <30, 30–44, 45–59, and 60+), which prevents us from exploring the differences within the group. For example, some countries’ definition of elderly is 65+, and the group 60+ mixes a group of 60–65-year-old mostly nonretired group, with the 65+ group of mostly retired. We further assess the impacts of carbon price on group 65+ with the Australian and US data (Fig. S4) thanks to data availability in these two countries. The results show that the group aged 65 and above remains to be the most affected by carbon pricing, which is consistent with our major finding. Nevertheless, the potential within-group difference deserves attention in future research. Third, due to the data availability, our study focuses on the impacts of carbon pricing on the elderly in developed countries. However, some studies pointed out that the elderly in developing countries have different consumption patterns from those in developed countries (58, 59). This requires more comprehensive data collection for future research.

## Supplementary Material

pgad209_Supplementary_Data

## Data Availability

The EXIOBASE 3.7 is available at https://www.exiobase.eu/ and https://zenodo.org/record/4588235#.YxoZS3bMKUk. Household expenditures by aging groups are sourced from the Household Budget Survey of EU (https://ec.europa.eu/eurostat/web/household-budget-surveys/database), Consumer Expenditure Survey of the United States (https://www.bls.gov/cex/csxstnd.htm), Family Income and Expenditure Survey of Japan (https://www.stat.go.jp/english/data/sousetai/1.html), and Household Budget Durvey of Australia (https://www.abs.gov.au/statistics/economy/finance/household-expenditure-survey-australia-summary-results). Household expenditures by income–age paired groups are sourced from the Consumer Expenditure Survey of the United States (https://www.bls.gov/cex/csxstnd.htm), Office for National Statistics of the United Kingdom (https://www.ons.gov.uk/peoplepopulationandcommunity/personalandhouseholdfinances/expenditure), and Family Income and Expenditure Survey of Japan (https://www.stat.go.jp/english/data/sousetai/1.html). Code to calculate the distribution impacts of global carbon price on age groups is available at https://github.com/PeipeiTian/Aging-society-and-carbon-pricing.
