# Supplementary material for: Implementation of carbon pricing in an aging world calls for targeted protection schemes
Source: PNAS Nexus. 2023 Jul 18;2(7):pgad209. doi: 10.1093/pnasnexus/pgad209 (PMC10353720; doi:10.1093/pnasnexus/pgad209)
Supplement: pgad209_Supplementary_Data [file pgad209_supplementary_data.docx]

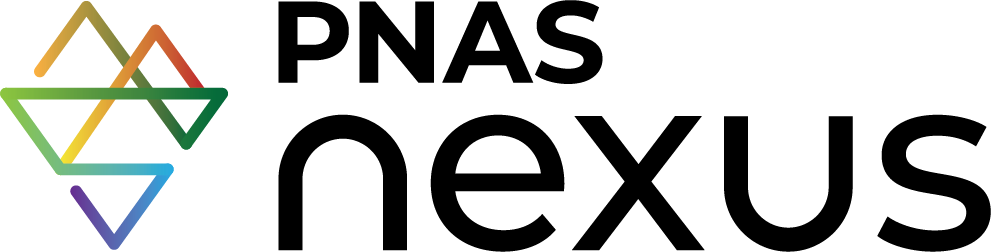


**Supporting Information for**

Implementation of carbon pricing in an aging world calls for targeted protection schemes

Peipei Tian, Kuishuang Feng, Heran Zheng, Klaus Hubacek, Jiashuo Li, Honglin Zhong, Xiangjie Chen, Laixiang Sun

* Corresponding author: Laixiang Sun, Honglin Zhong, Heran Zheng

**Email:** lsun123@umd.edu (Laixiang Sun), heran.zheng@ucl.ac.uk (Heran Zheng), honglin.zhong@sdu.edu.cn (Honglin Zhong)

**This PDF file includes:**

Supporting text

Figures S1 to S4

SI References

Supporting Information Text

**Matching the household expenditure survey data to EXIOBASE**

To capture the heterogeneity of the impacts of carbon pricing on different income and age groups, household demand vectors of studied countries in EXIOBASE are disaggregated by age and income groups based on the household expenditure survey (HES) data. We use the classical RAS-based method to bridge the difference (including classification and price differences) between the HES data and Input-output table in EXIOBASE (1, 2). Specifically, we developed a bridging matrix that links the expenditure in sectors of goods and services of the HES data to the corresponding 200 sectors of EXIOBASE for every country individually. In the reconciliation, EXIOBASE’s household demand is set as the benchmark and HES data as balance constraints (3, 4) (Fig. S1). We use the relative total expenditure and expenditure structure information for each age group in the HES data to adjust the final demand of EXIOBASE. The reconciliation is always going on under the basic prices. Finally, we get household final demand consistent with EXIOBASE classification of different income and age groups in study countries.

It is worth noting that the household or consumer expenditure survey (HES) data of 27 EU and the UK are presented in four age groups: aged <30, aged 30-44, aged 45-49, and aged 60 and over, while the US and Australia have their age classification with six age groups: aged <25, aged 25-34, aged 35-44, aged 45-54, aged 55-64, and aged 65 and over. Therefore, to facilitate the expression, we combine the modelled impacts of carbon pricing of six age groups of the US and Australia into four age groups to be compatible with EU countries and the UK. Notably, when reconciling the HES data into EXIOBASE, we keep their own categorisation to avoid uncertainty of aggregation. In other words, the household consumption for the US and Australia in the EXIOBASE is disaggregated into six groups, while the household consumption of EU countries and the UK are disaggregated into four groups. Therefore, we can get impacts of carbon pricing of four age groups for EU countries and the UK, and six age groups for the US and Australia. Then, the results of the US and Australia are combined into four age groups (due to no data about the distribution of impacts of carbon pricing or population in a given age group, we then assume the even distribution of impacts of carbon pricing or population in a given age group(4)).

For Australia and the US:

The aged <30 = aged<25+1/2 *aged 25-34;

The aged 30-44 = 1/2 *aged 25-34+ aged 35-44;

The aged 45-60 = aged 45-54 + 1/2 *aged 55-64;

The aged <60 = 1/2 *aged 55-64+65 and over

The HES data of the income-age paired groups in the US, the UK, and Japan are matched to EXIOBASE in the same way as in the case of the age group. Specifically, the household demand vectors of every age group are further disaggregated by income groups based on HES data (Fig. S1 b). The HES data of the income groups of the US is presented as specific amounts: household income <$5000, household income $5000-$10000, household income $10000-$15000, household income $15000-$20000, household income $20000-$30000, household income $30000-$40000, household income $40000-$50000, household income $50000-$70000, and household income >$70000. Considering the population size of each group, we combine the results into four income groups (household income< $20000, household income $20000-$40000, household income $40000-$70000, and household income >$70000) based on the assumption of even distribution mentioned above. For the UK and Japan, the income groups are presented ad quintiles in the HES data. Notably, the 2015 HES data of income-age paired groups in Japan is unavailable, we thereby match the 2014 HES data of income-age paired groups to 2015 EXIOBASE.

**Design options for carbon pricing**

Although the global carbon price is regarded as economically optimal for climate change mitigation, it is hard to come true so soon as being constrained by economic and political realities(5, 6). By contrast, national and sector’s carbon prices have been implemented in many countries(7, 8). Therefore, we further devise three carbon price scenarios to discuss the impacts of carbon pricing on younger and elderly groups. 1) global carbon price (GCP) scenario, where the carbon pricing is implemented in all countries around the world, which implies that all carbon emissions in every country will be affected by the carbon pricing; 2) National carbon price (NCP) scenario. The NCP scenario refers to that the carbon pricing policy is only implemented in own country, which indicates that only national carbon emissions are considered in NCP. For example, in terms the US, the NCP of the US means that only the carbon emission in the US is affected by the carbon pricing, while carbon emission in other countries is not affected by the carbon pricing. Notably, due to the unity of carbon policies and economic activities in EU countries, the NCP for every EU country implies that the carbon emission in all EU countries is affected by the carbon pricing. 3) Electric sector carbon price (ECP) scenario, where the national carbon prices are applied to the power sector only. Three carbon price scenarios are only different in terms of carbon emission coverage, and all carbon price is set at $40 per tCO_2_.

Figure S2 shows that the increase rate in per capita expenditure of elderly groups under national and electric sector carbon prices remains higher than that of younger groups. Notably, despite the absolute impacts of carbon pricing on the elderly group becoming smaller due to the reduced coverage of carbon emission under NCP and ECP scenarios, the distributional inequalities between younger and elderly groups induced by carbon pricing are more serious in most countries, especially in western EU countries. Compared to the global carbon price scenario, the relative disparity in the impacts of the ECP scenario between elderly and younger groups expands by more than 10% in many richer countries (e.g., western EU countries, the UK and Japan) and reaches 20% under ECP scenario. For example, the expenditure rise rates of the elderly group in Finland are 35% and 17% higher than that of younger groups with the ECP and NCP scenarios, respectively, while this number is 12% under the GCP scenario. Multi scenario analysis shows that carbon pricing has the widespread unequal impacts between younger and elderly groups.

**
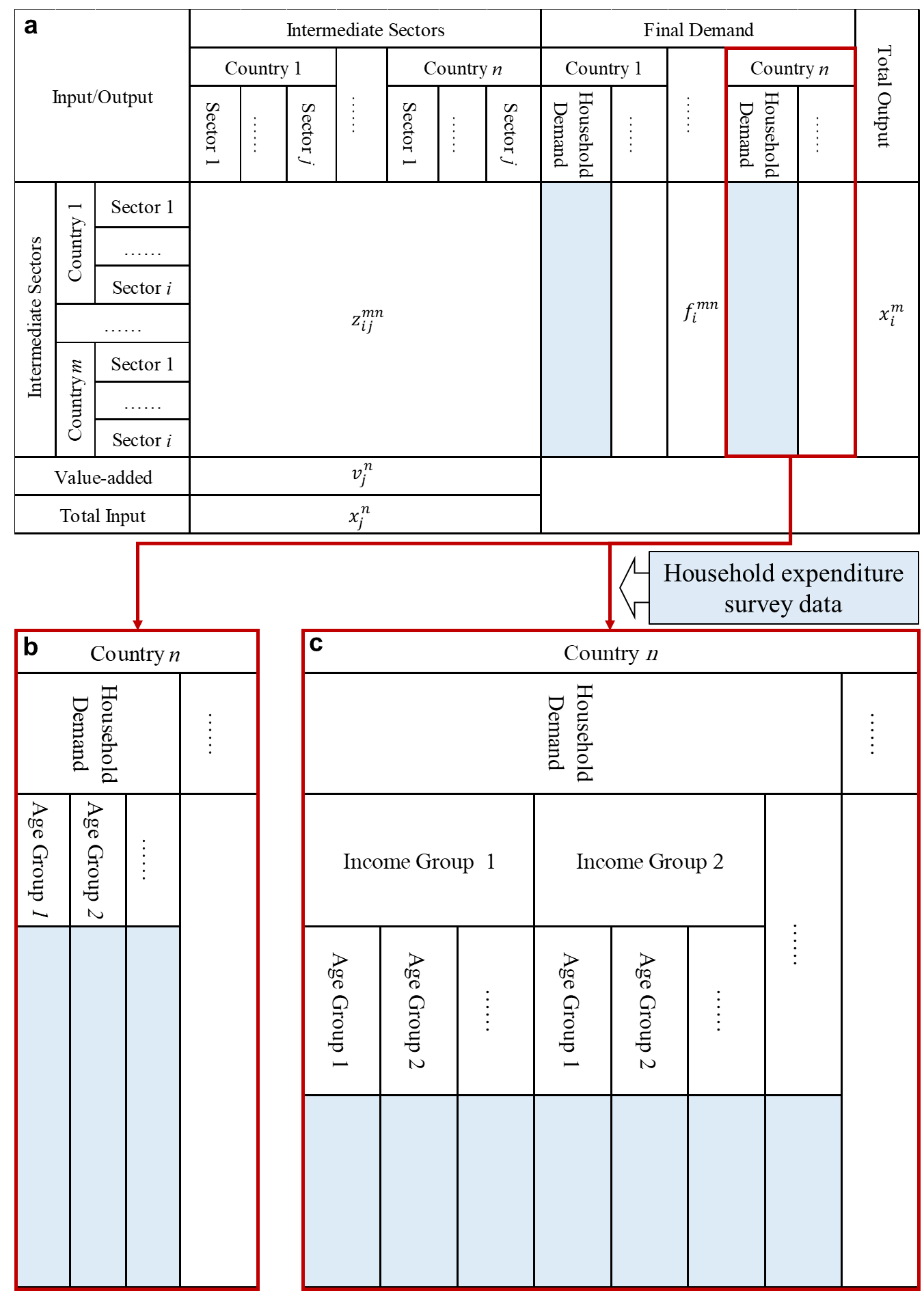
**

**Fig. S1. The schematic of matching the household expenditure survey data to EXIOBASE.** **a,** The MRIO table of EXIOBASE. **b,** The disaggregation by age groups of household demand. **c,** The disaggregation by age and income groups of household demand.


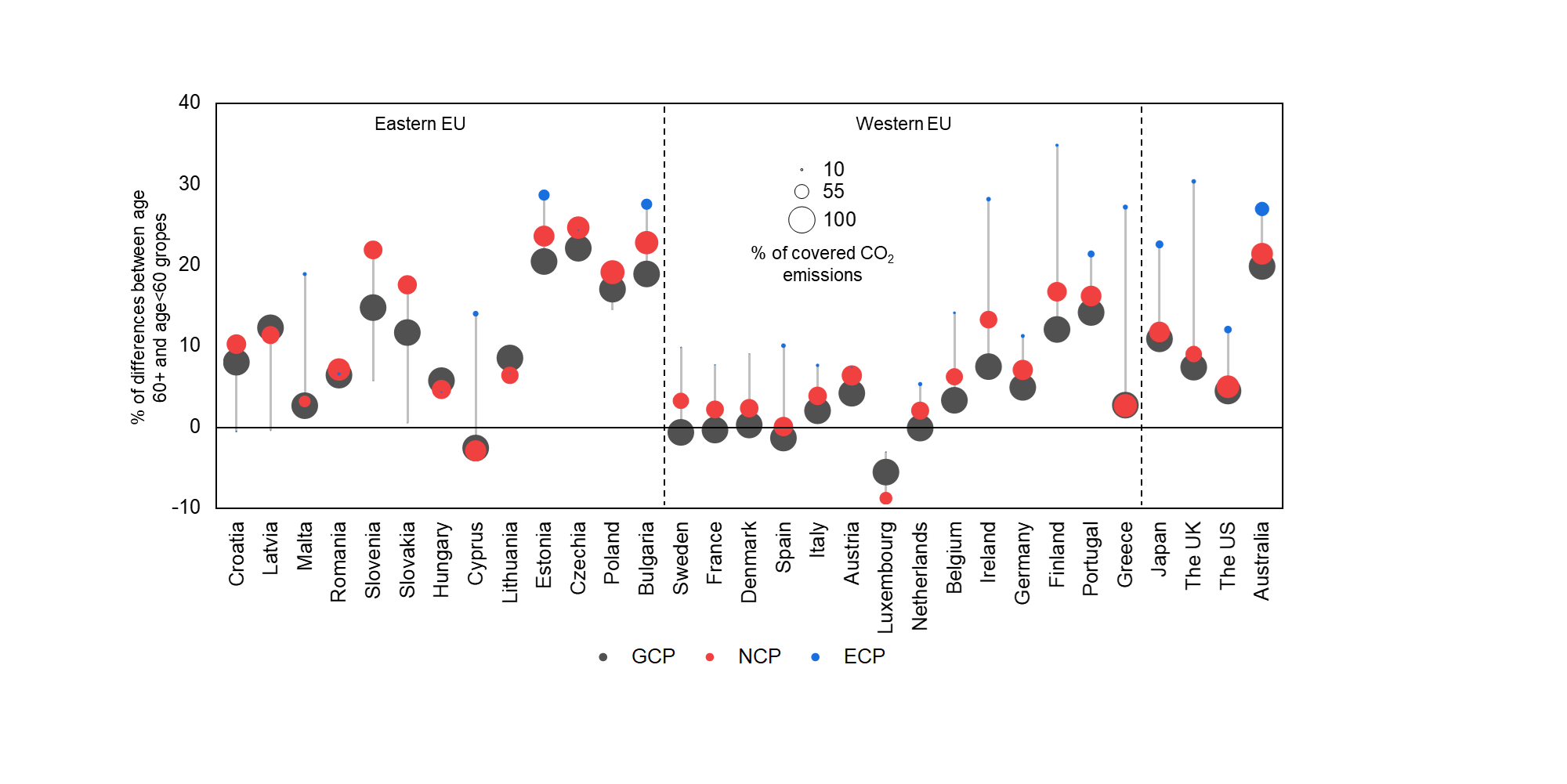


**Figure S2 The relative disparities in the impact of three carbon price scenarios on 60+ and 60- groups.** GCP, NCP, and ECP refer to global, national, and electric industry carbon pricing scenarios, respectively. The sizes of the dots refer to the proportion of carbon emissions covered by the carbon price scenarios.


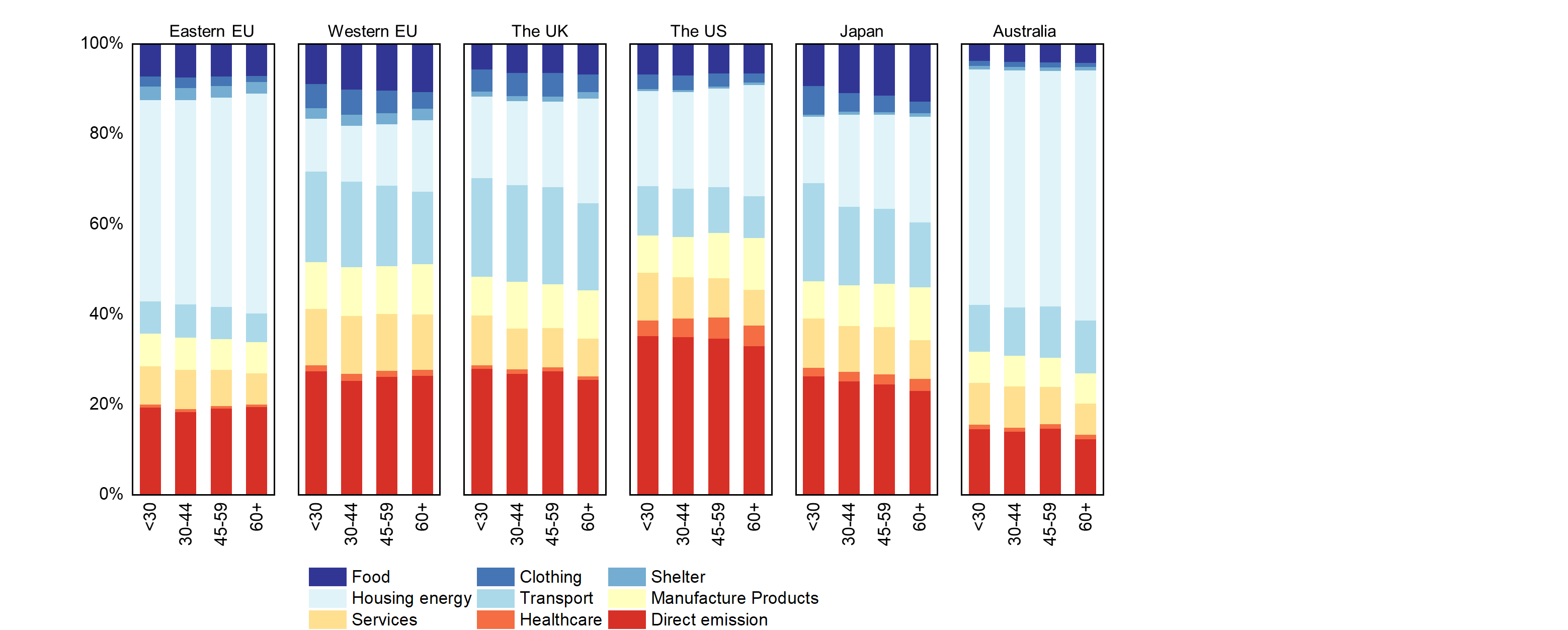


**Figure S3 Sectoral structure of additional expenditures under $40 per t CO_2_ global carbon price for four age groups in 31 northern countries.**

**
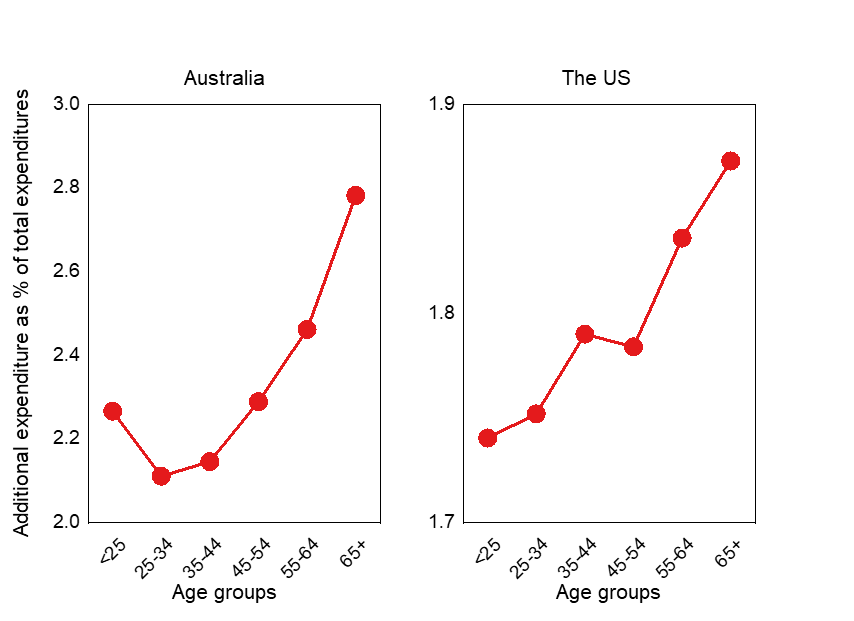
**

**Figure S4 Impacts of a $40 per t CO_2_ global carbon price on the age groups in Australia and the US.**

**SI References**

1. Y. Oswald, A. Owen, J. K. Steinberger, Large inequality in international and intranational energy footprints between income groups and across consumption categories. *Nat. Energy* **5**, 231–239 (2020).

2. R. E. Miller, P. D. Blair, *Input-output analysis: foundations and extensions* (Cambridge university press, 2009).

3. K. Steen‐Olsen, R. Wood, E. G. Hertwich, The carbon footprint of Norwegian household consumption 1999–2012. *J. Ind. Ecol.* **20**, 582–592 (2016).

4. H. Zheng, *et al.*, Ageing society in developed countries challenges carbon mitigation. *Nat. Clim. Chang.* **12**, 241–248 (2022).

5. O. Edenhofer, *et al.*, Closing the emission price gap. *Glob. Environ. Chang.* **31**, 132–143 (2015).

6. J. C. Steckel, *et al.*, Distributional impacts of carbon pricing in developing Asia. *Nat. Sustain.* (2021) https:/doi.org/10.1038/s41893-021-00758-8.

7. M. Hintermayer, A carbon price floor in the reformed EU ETS: Design matters! *Energy Policy* **147**, 111905 (2020).

8. M. Santikarn, *et al.*, State and trends of carbon pricing 2021 (2021).
